# Unintended Pregnancy and Abortion in the US Navy, 2016

**DOI:** 10.1007/s11606-022-07582-6

**Published:** 2022-08-31

**Authors:** Kate Grindlay, Jane Seymour, Laura Fix, Daniel Grossman

**Affiliations:** 1 Ibis Reproductive Health, Cambridge, MA USA; 2 Advancing New Standards in Reproductive Health, Bixby Center for Global and Reproductive Health, Department of Obstetrics, Gynecology and Reproductive Sciences, University of California, San Francisco, Oakland, CA USA

**Keywords:** abortion, military personnel, navy personnel, pregnancy, pregnancy, unplanned, reproductive health, women

## Abstract

**Background:**

The unintended pregnancy rate in the US military is higher than among civilians. While 42% of unintended pregnancies end in abortion among civilian women, there are no data on the prevalence of abortion in the military overall or by service branch.

**Objective:**

This analysis was conducted to estimate unintended pregnancy rates and the percentage of unintended pregnancies that resulted in abortion among active-duty US Navy members aged 44 years or younger reporting female gender in 2016.

**Design:**

Cross-sectional survey data from the 2016 Navy Pregnancy and Parenthood Survey, collected from August to November 2016.

**Participants:**

Our sample included 3,423 active-duty US Navy members aged 44 years or younger reporting female gender, generated from a stratified random sample of 38% of all active-duty Navy women in pay grades E2-E9 and O1-O5 in 2016; the survey had a 20% response rate for females.

**Main Measures:**

We calculated pregnancy and unintended pregnancy rates, the percentage of pregnancies that were unintended, and the percentage of unintended pregnancies resulting in birth and abortion in the prior fiscal year.

**Key Results:**

Overall, the self-reported unintended pregnancy rate was 52 per 1,000 participants and 38.1% of pregnancies were unintended. The adjusted unintended pregnancy rate accounting for abortion underreporting was 68 per 1,000 participants. Unintended pregnancy rates were highest among individuals who were younger (aged 18–24) and in enlisted pay grades, compared to their counterparts. Six percent reported their unintended pregnancy resulted in abortion. Six respondents reported becoming pregnant while deployed; none of these pregnancies resulted in abortion.

**Conclusions:**

In this first study to report on abortion prevalence among US servicemembers, we found the proportion of unintended pregnancies resulting in abortion among a sample of US Navy members in 2016 was much lower than civilians, yet unintended pregnancy rates were higher.

## INTRODUCTION

An unintended pregnancy is one that occurs when someone wants to become pregnant in the future but not at the time they became pregnant or when they do not want to become pregnant then or at any time in the future.^1^ Categorizing pregnancy desires is complex and current measurements of unintended pregnancy have limitations, which have led researchers to explore new ways of assessing pregnancy intention.^1–4^ Regardless of these measurement limitations, births resulting from unintended pregnancies are associated with negative maternal and child health outcomes,^5,6^ and unintended pregnancy remains a widely used metric for benchmarking health promotion and disease prevention.^7^

Unintended pregnancy has been shown to be higher in the United States (US) military population compared to the civilian population. In analyses of data from the Department of Defense’s Health Related Behaviors Survey, a representative survey of active-duty military personnel conducted every 2–4 years, the unintended pregnancy rate among active-duty servicewomen aged 18–44 years was 72 per 1,000 women in 2011.^8^ Summary reports from the subsequent rounds of the survey from 2015 and 2018 reported 4.8% and 5.5% of military women, respectively, experienced an unintended pregnancy in the prior year; however, these findings were not restricted to servicewomen of reproductive age, aged 44 years or younger, precluding direct comparison to the prior survey rounds or the civilian population.^9,10^ The 2011 Health Related Behaviors Survey found that women in the Navy had among the highest rates of unintended pregnancy among the service branches, at 95 per 1,000 women.^8^ The latest unintended pregnancy rate among civilian women, from 2011, was 45 per 1,000 women aged 15–44 years.^11^

Official US Navy guidance explicitly aims to accommodate work-life balance for parents in Navy service and stipulates that, to the greatest extent possible, servicemembers’ careers should not be negatively impacted by pregnancy.^12^ Following a birth, Navy members are allowed 6 weeks of maternity convalescent leave and 6 weeks primary caregiver leave, as well as 2 weeks of secondary caregiver leave, under Military Parental Leave Program.^12^ While this leave is more than many employers offer in the USA, the Defense Advisory Committee on Women in the Services, a Department of Defense federal advisory committee appointed by the Secretary of Defense to provide advice and recommendations related to servicewomen in the Armed Forces, states this policy “falls short of mitigating the considerable physical and mental challenges [servicemembers] face trying to achieve a reasonable work-life balance” and that work-life balance challenges are further exacerbated by deployments, repeated moves, and distance from family support systems.^13^ Because pregnant servicemembers cannot remain in certain military positions or roles, they lose work time during and after pregnancy, and must also be evacuated if deployed and returned to their home station.^14^ Prior research has found concerns among servicemembers that pregnancy and associated stigmas, lost work time, and deployment disruptions could harm their careers,^15–18^ despite official guidance that pregnancy will not be the basis for downgrading marks or adverse career impacts.^12^

For servicemembers seeking to terminate a pregnancy, federal law only allows abortion provision and TRICARE insurance coverage in cases of rape, incest, and life endangerment of the pregnant person.^19^ Most servicemembers must therefore seek abortion care outside the military health care system. Research has found numerous barriers to servicemembers seeking abortion care on their own, including legal, logistical, and financial barriers, concerns about confidentiality, and fear of negative career impacts.^15–17^ In the civilian population, the percentage of unintended pregnancies that resulted in abortion was 42% in 2011.^11^ There are no published data on the prevalence of abortion among US servicemembers with unintended pregnancies.

The Navy Pregnancy and Parenthood Survey, conducted biennially since 1988, is designed to collect data for evaluating pregnancy policies on parenthood and pregnancy for the Department of the Navy,^20^ and some survey rounds include a question on pregnancy outcome, including abortion. This analysis was conducted to estimate unintended pregnancy rates and the percentage of unintended pregnancies that resulted in abortion among active-duty US Navy members aged 44 years or younger reporting female gender in 2016.

## MATERIALS AND METHODS

### Data Source

Cross-sectional data from the 2016 Navy Pregnancy and Parenthood Survey were used to calculate pregnancy and unintended pregnancy rates, the percentage of pregnancies that were unintended, and the percentage of unintended pregnancies that resulted in birth and abortion in the prior fiscal year among active-duty US Navy members aged 44 years or younger reporting female gender. The survey was conducted by the Naval Air Warfare Center Training Systems Division (NAWCTSD) Air Branch 4635 (Manpower and Personnel Studies).^20^ The data used for this analysis were obtained via Freedom of Information Act request from the Naval Air Warfare Center.

### Survey Design, Sampling, and Data Collection

Data were collected from August to November 2016. The target population included a stratified random sample of 22,924 Navy women (38% of all active-duty Navy women) and 9,665 Navy men (4% of all active-duty Navy men) in pay grades E2-E9 and O1-O5. Participants received a survey invitation letter and two reminder letters at their command address and three reminder emails. The survey was conducted online. Overall, 4,802 participants reporting male or female gender completed the survey, with a 20% return rate for females after correcting the sample size for return-to-sender errors.^20^

The survey asked a common core set of questions to all participants on retention influencers, parenthood, family planning, sabbaticals, attitudes toward birth control and health care providers, and adoption leave. To maintain anonymity of respondents, the survey manager removed all login information from the data before analysis^20^ and this information was not included in our dataset.

### Inclusion Criteria

We restricted our sample to participants of reproductive age, aged 44 years or younger, reporting female gender. Gender was assessed by the question, “What is your gender?” from which respondents could select “male” or “female.”

### Outcomes

Our outcomes of interest were unintended pregnancy and unintended pregnancy that resulted in abortion in the prior fiscal year. Pregnancy in the prior fiscal year was measured by answering “yes” to the question, “Did you become pregnant between 1 October 2014 and 30 September 2015? (Do NOT count pregnancies that began before 1 October 2014 even though you were pregnant on that date.).” One additional participant was coded as having a pregnancy during the prior fiscal year as they reported a pregnancy in that time period in response to a question about how long ago their most recent pregnancy during Navy service occurred.

Unintended pregnancy in the prior fiscal year was measured by answering “yes” to the question, “Did you have an UNPLANNED pregnancy between 1 October 2014 and 30 September 2015? Note: For this survey, a planned pregnancy is one that you wanted at that time (i.e., you intentionally became pregnant.).”

Participants were also asked a series of questions about their most recent pregnancy since entering the Navy, including on the pregnancy outcome, whether they were deployed when they became pregnant, and whether they were using birth control at the time of the pregnancy and if not, why not. We restricted analyses of these variables to participants who reported an unintended pregnancy in the prior fiscal year and assumed that reporting on most recent pregnancy among these individuals referred to the pregnancy in the prior fiscal year.

The outcomes of participants’ unintended pregnancies in the prior fiscal year (abortion or birth) were assessed by the question, “What was the outcome of [your most recent] pregnancy?” We excluded participants who reported their unintended pregnancy outcome as currently pregnant, having had a miscarriage, or having had an ectopic pregnancy so as to include pregnancy outcomes determined by the participant. Participants were coded as having had an abortion if they reported their most recent pregnancy outcome as “abortion.” Participants were coded as having had a birth if they reported their most recent pregnancy outcome as “live birth (delivery after 36th week of gestation),” “premature birth (delivery between the 20th through 36th week of gestation),” or “stillbirth.”

### Covariates

Participants were asked demographic questions on their current age, marital status, and pay grade, as well as their deployment status when they became pregnant; race and ethnicity questions were not asked in the survey. Covariates were selected based on availability in the dataset, associations with unintended pregnancy found in prior research with servicemembers,^8,21,22^ and a priori interest in the impacts of deployment status on pregnancy outcomes. We coded participants’ pay grades as enlisted (E1-9) or officer (W2-5, O1-O7 or above); two respondents did not answer this question; however, a variable for Navy rate that was included in the dataset indicated they were in the enlisted pay grade and we coded them accordingly. Deployment at the time of unintended pregnancy was measured by answering “deployed” to the question, “Where was your [Navy] unit in the operational cycle when you [most recently] became pregnant?”

### Data Analyses

Descriptive statistics were calculated with Stata Statistical Software version 15.1 (StataCorp, College Station, TX). Missing data (nine participants who did not respond to the questions on prior pregnancy; one missing entry for two covariates; one missing entry for birth control use at time of most recent pregnancy) were excluded and the sample size for our outcomes of interest varied based on item response and survey skip patterns. The STROBE guidelines were used in reporting our study findings.^23^ This study was approved by the Allendale Investigational Review Board.

For the prior fiscal year overall and by background characteristics, we calculated the total number of pregnancies, the number of pregnancies that were unintended, and the percentage of pregnancies that were unintended. We divided the number of pregnancies and unintended pregnancies by the total sample population to obtain self-reported pregnancy and unintended pregnancy rates per 1,000 participants, by participants overall and by available background characteristics, including age group (18–24, 25–29, 30–44), relationship status (single, never married; divorced, widowed, separated; married), and pay grade (enlisted, officer); 95% confidence intervals (CIs) were also calculated for unintended pregnancy rates overall and by background characteristics.

We also calculated adjusted unintended pregnancy rates to account for underreporting of abortion. To do this, we employed methodology previously used to measure unintended pregnancy in the civilian population and the US military.^22^ Using this methodology, we increased the self-reported number of pregnancies reported by 11.9% (to mirror household survey findings that nearly half of abortions are underreported, reflecting 11.9% of the total number of reported pregnancies); 95% of these additional pregnancies were assumed to be unintended, as they represented induced abortions.^22^ We added these additional unintended pregnancies to the unintended pregnancies that were self-reported to calculate the adjusted unintended pregnancy rate.

Finally, we calculated the percentage of self-reported unintended pregnancies in the prior fiscal year that resulted in birth and abortion, along with 95% CIs for these overall and by background characteristics, including age group, relationship status, pay grade, and deployment status at the time of the pregnancy.

## RESULTS

After excluding those who did not identify their gender as female (*n*=1,165) and who were over the age of 44 (*n*=214), our sample included 3,423 participants. Most participants were aged 30–44 years (range: 19–44 years), married, and in the enlisted pay grade. Overall, the unintended pregnancy rate was 52 per 1,000 participants (95% CI: 45–60) and 38.1% of pregnancies (95% CI: 33.8–42.6%) were unintended in 2016. The adjusted unintended pregnancy rate accounting for abortion underreporting was 68 per 1,000 participants. Unintended pregnancy rates were highest among younger individuals aged 18–24 and enlisted individuals, compared to their counterparts. (See Table 1.)

**Table 1:** Pregnancy Rates and Percentage That Were Unintended in the Prior Fiscal Year, Among US Navy Members Aged 44 Years or Younger Reporting Female Gender in 2016 (*N*=3,414)

Characteristic	All participants	Number of pregnancies		Percentage of pregnancies that were unintended	Pregnancy rate per 1,000 servicemembers		
	*n* (%)	Self-reported total	Self-reported unintended	%	Self-reported total	Self-reported unintended	Adjusted unintended^*^
All females	3,414 (100%)	467	178	38.1%	137	52 (95% CI: 45–60)	68
Age group (in years)							
18–24	729 (21%)	75	52	69.3%	103	71 (95% CI: 55–92)	83
25–29	926 (27%)	119	56	47.1%	129	60 (95% CI: 47–78)	75
30–44	1,759 (52%)	273	70	25.6%	155	40 (95% CI: 32–50)	57
Relationship status							
Single, never married	1,205 (35%)	65	49	75.4%	54	41 (95% CI: 31–53)	47
Divorced, widowed, separated	422 (12%)	37	30	81.1%	88	71 (95% CI: 50–100)	81
Married	1,786 (52%)	364	98	26.9%	204	55 (95% CI: 45–66)	78
Pay grade							
Enlisted	1,945 (57%)	279	152	54.5%	143	78 (95% CI: 67–91)	94
Officer	1,469 (43%)	188	26	13.8%	128	18 (95% CI: 12–26)	32

Columns may not tally to 100% owing to missing values

^*^Adjusted for abortion underreporting

*CI* confidence interval

Of the 178 participants who reported an unintended pregnancy in the prior fiscal year, 150 reported their pregnancy outcome as birth or abortion and were included in our analyses of pregnancy outcomes (excluded from this analysis were two who reported ectopic pregnancy, eight who were still pregnant, and 18 who reported miscarriage). Among these 150 respondents with an unintended pregnancy in the prior fiscal year, only nine (6.0%, 95% CI: 3.1–11.2%) reported that the pregnancy resulted in abortion. No unintended pregnancies resulting in abortion were reported by individuals who were deployed when they became pregnant. (See Table 2.)

**Table 2:** Percentage of Self-reported Unintended Pregnancies in the Prior Fiscal Year That Resulted in Birth and Abortion, Among US Navy Members Aged 44 Years or Younger Reporting Female Gender in 2016 (*N*=150)

	All self-reported unintended pregnancies^*^	Unintended pregnancies that resulted in birth^†^	Unintended pregnancies that resulted in abortion
		*n* (%)	*n* (%)
All females	150	141 (94.0%, 95% CI: 88.8–96.9%)	9 (6.0%, 95% CI: 3.1–11.2%)
Age group (in years)			
18–24	42	41 (97.6%, 95% CI: 84.7–99.7%)	1 (2.4%, 95% CI: 0.3–15.3%)
25–29	52	47 (90.4%, 95% CI: 78.8–96.0%)	5 (9.6%, 95% CI: 4.0–21.2%)
30–44	56	53 (94.6%, 95% CI: 84.5–98.3%)	3 (5.4%, 95% CI: 1.7–15.5%)
Relationship status			
Single, never married	35	30 (85.7%, 95% CI: 69.8–94.0)	5 (14.3%, 95% CI: 6.0–30.2%)
Divorced, widowed, separated	27	25 (92.6%, 95% CI: 74.5–98.2%)	2 (7.4%, 95% CI: 1.8–25.5%)
Married	87	86 (98.9%, 95% CI: 92.2–99.8%)	1 (1.2%, 95% CI: 0.2–7.8%)
Pay grade			
Enlisted	126	119 (94.4%, 95% CI: 88.7–97.3%)	7 (5.6%, 95% CI: 2.7–11.3%)
Officer	24	22 (91.7%, 95% CI: 71.9–97.9%)	2 (8.3%, 95% CI: 2.1–28.1%)
Deployed at time of pregnancy			
Yes	6	6 (100.0%)	0 (0.0%)
No	133	126 (94.7%, 95% CI: 89.3–97.5%)	7 (5.3%, 95% CI: 2.5–10.7%)

Columns may not tally to 100% owing to missing values

^*^Pregnancies that resulted in miscarriage or ectopic pregnancies as well as people who were still pregnant were excluded

^†^Birth included live birth, premature birth, and still birth

*CI* confidence interval

Sixty percent of participants with an unintended pregnancy in the prior fiscal year (106/177) reported they were not using contraception at the time of their unintended pregnancy. Among these participants (*n*=106), 27.4% reported they or their partner did not want to use birth control; 17.0% thought they or their partner was infertile; 3.8% were not comfortable discussing or getting birth control, and 2.8% cited personal or religious beliefs; 49.1% reported some other reason for not using contraception at the time of pregnancy (the data on other reasons were not included in our dataset). Data not shown.

## Discussion

In this first study to report on abortion prevalence among US servicemembers, we found that while unintended pregnancy rates among this sample of US Navy servicemembers in 2016 remained higher than in the civilian population, the proportion of self-reported unintended pregnancies resulting in abortion was much lower (6.0%) compared to civilians (42%^11^).

The lower proportion of unintended pregnancies resulting in abortion in this sample compared to civilians may in part indicate that US servicemembers face increased barriers to abortion access owing to restrictions under federal law, or that these restrictions resulted in fewer Navy members seeking terminations compared to people in the general US population. It may also be possible that lower abortion reporting in this sample is a result of underreporting, especially in the context of the military, where abortion is highly stigmatized. The lower proportion of abortions in this sample may also reflect differing attitudes toward abortion in the military compared to the civilian population; however, a cross-sectional online survey among a convenience sample of current and former servicewomen found 81% reported they could accept someone’s decision to end a pregnancy, and the majority believed the military should cover and provide abortion for unwanted pregnancies.^18^ A 2014–2016 study comparing rates of induced abortion among veterans receiving Veterans Affairs healthcare to rates in the general US population found that while veterans had a lower proportion of unintended pregnancies that resulted in abortion in the past year (9.8%) compared to civilians (18.3%), veterans were more likely than civilians to report ever having an abortion,^24^ highlighting the relevance and utilization of abortion care for this population.

The unintended pregnancy rate overall in this study, 52 per 1,000 participants, is lower than the rate among Navy women from the most recent Health Related Behaviors Survey for which data are available (from 2011), 95 per 1,000 participants; however, both surveys follow a similar trend in having higher unintended pregnancy rates among enlisted servicemembers compared to officers.^8^ While the methodology between the two surveys differs, the lower unintended pregnancy rate in the 2016 Navy Pregnancy and Parenthood Survey may in part reflect efforts that have taken place during the intervening time in the Navy and Marine Corps and military overall to expand access to contraception, which have been shown to increase contraceptive use and use of long-acting reversible contraceptives among servicemembers.^25,26^ Between 2012 and 2016, long-acting reversible contraceptive use increased among active-duty members of the US military from 17 to 22%, which may be attributed to educational and other programs such as walk-in clinics facilitating greater access.^27^ While the unintended pregnancy rate may be decreasing in the Navy, it appears to remain higher than the civilian rate, 45 per 1,000 women.^11^

This study has several limitations. While we attempted to account for abortion underreporting, the prevalence of unintended pregnancy and abortion may still be underreported owing to their sensitivity. While the methodology we employed for adjusting for abortion underreporting has been used previously for a military population,^22^ it is possible that extrapolating underreporting from US nationally representative household surveys undercounts true abortion underreporting in the military context. Additionally, the survey only included active-duty participants, and therefore some Navy members who became pregnant in the prior year may not have been eligible to participate if they were no longer on active-duty service as a result of their pregnancy, further underestimating pregnancy, unintended pregnancy, and abortion. The sole use of female/male response options in the first question of the survey on gender may also have deterred people who have experienced pregnancy but who hold a gender identity other than female, or who have an intersex experience, from participating in the survey. While we use “female” in this paper for participants as it reflects the language used in the dataset, we acknowledge that people with gender identities beyond “female” or “women” can and do experience pregnancy and abortion care.^28^ In the 2016 Workplace and Gender Relations Survey of Active Duty Members conducted by the Department of Defense, 1,850 active-duty servicemembers (among 126,234 active-duty participants overall) identified as transgender men, assigned female at birth.^29^ Additionally, given the low response rate for the survey, there is the potential for selection bias. The 2016 Navy Pregnancy and Parenthood Survey researchers statistically weighted results by pay grade and gender strata to be representative of the entire Navy population at the time of survey administration; however, we did not receive the survey weights in our dataset and therefore our analyses reflect the survey population and is not necessarily representative of the Navy as a whole. We also did not receive open-response questions in our dataset, limiting our ability to analyze some data.

## Conclusions

This study suggests that abortion in the military may be much lower than in the civilian population, despite unintended pregnancy being higher. More research is needed to understand the underlying factors and the extent to which federal legal restrictions to abortion access for servicemembers may play a role.
